# The novel TRAIL-receptor agonist APG350 exerts superior therapeutic activity in pancreatic cancer cells

**DOI:** 10.1038/s41419-018-0478-0

**Published:** 2018-04-18

**Authors:** Karen Legler, Charlotte Hauser, Jan-Hendrik Egberts, Anna Willms, Carola Heneweer, Susann Boretius, Christoph Röcken, Claus-Christian Glüer, Thomas Becker, Michael Kluge, Oliver Hill, Christian Gieffers, Harald Fricke, Holger Kalthoff, Johannes Lemke, Anna Trauzold

**Affiliations:** 1 Institute for Experimental Cancer Research, Christian-Albrechts-University of Kiel, Kiel, Germany; 2 Clinic for General Surgery, Visceral, Thoracic, Transplantation and Pediatric Surgery, University Hospital Schleswig-Holstein, Kiel, Germany; 3 Clinic for Diagnostic Radiology and Neuroradiology, University Hospital Schleswig-Holstein, Kiel, Germany; 4 Department of Radiology, University Hospital Cologne, Cologne, Germany; 5 Functional Imaging Laboratory, German Primate Center, Leibniz Institute for Primate Research and Georg-August-University Göttingen, Göttingen, Germany; 6 Institute of Pathology, University Hospital Schleswig-Holstein, Kiel, Germany; 7 Section Biomedical Imaging, Department of Diagnostic Radiology und Neuroradiology, University Hospital Schleswig-Holstein, Kiel, Germany; 8 APOGENIX AG, Im Neuenheimer Feld 584, Heidelberg, Germany; 9 Affimed GmbH, Im Neuenheimer Feld 582, Heidelberg, Germany; 10 Clinic of General and Visceral Surgery, University Hospital Ulm, Ulm, Germany

## Abstract

Tumor necrosis factor-related apoptosis-inducing ligand (TRAIL) has raised attention as a novel anticancer therapeutic as it induces apoptosis preferentially in tumor cells. However, first-generation TRAIL-receptor agonists (TRAs), comprising recombinant TRAIL and agonistic receptor-specific antibodies, have not demonstrated anticancer activity in clinical studies. In fact, cancer cells are often resistant to conventional TRAs. Therefore, in addition to TRAIL-sensitizing strategies, next-generation TRAs with superior apoptotic activity are warranted. APG350 is a novel, highly potent TRAIL-receptor agonist with a hexavalent binding mode allowing the clustering of six TRAIL-receptors per drug molecule. Here we report on preclinical in vitro and in vivo studies testing the activity of APG350 on pancreatic ductal adenocarcinoma (PDAC) cells. We found that APG350 potently induced apoptosis of Colo357, PancTuI and Panc89 cells in vitro. In addition, APG350 treatment activated non-canonical TRAIL signaling pathways (MAPK, p38, JNK, ERK1/ERK2 and NF-κB) and induced the secretion of IL-8. Stable overexpression of Bcl-xL inhibited APG350-induced cell death and augmented activation of non-canonical pathways. Intriguingly, pre-treatment of Bcl-xL-overexpressing cells with the BH3-mimic Navitoclax restored their sensitivity to APG350. To study the effects of APG350 on PDAC cells in vivo, we applied two different orthotopic xenotransplantation mouse models, with and without primary tumor resection, representing adjuvant and palliative treatment regimes, respectively. APG350 treatment of established tumors (palliative treatment) significantly reduced tumor burden. These effects, however, were not seen in tumors with enforced overexpression of Bcl-xL. Upon primary tumor resection and subsequent APG350 treatment (adjuvant therapy), APG350 limited recurrent tumor growth and metastases. Importantly, therapeutic efficacy of APG350 treatment was more effective compared with treatment with soluble TRAIL in both models. In conclusion, APG350 represents a promising next-generation TRA for the treatment of PDAC. Moreover, our results suggest that combining APG350 with Navitoclax might be a succesfull strategy for cancers harboring mitochondrial apoptosis resistance.

## Introduction

Despite tremendous progress in molecular and clinical oncology, pancreatic ductal adenocarcinoma (PDAC) still remains a devastating disease with 5-year-survival rates of only about 5%^1^. For many decades, it is the fourth/fifth leading cause of cancer death, and predicted to become the second in 2030 in the United States^2^. Several reasons account for these alarming figures. First, PDAC cells tend to exhibit early invasive growth into neighboring tissue and systemically spread to lymph nodes and other organs, most importantly the liver. Second, unspecific and vague symptoms often delay the diagnosis of PDAC. Third, PDAC cells are widely resistant to conventional radio- and chemotherapy^3^. Thus, novel therapeutic strategies are urgently needed for this malignancy.

The death ligand tumor necrosis factor (TNF)-related apoptosis-inducing ligand (TRAIL) was identified due to its sequence homology with TNFα and CD95L/FASL^4,5^. TRAIL is capable of inducing apoptotic cell death via binding to its two membrane-bound receptors TRAIL-R1 and TRAIL-R2^6,7^. Upon receptor triggering, the formation of the death-inducing signaling complex (DISC) is initiated. Within the DISC, the adapter protein FADD is recruited, which in turn leads to recruitment and activation of caspases-8 and/or -10 ^8^. In type-I cells, the level of activated caspase-8/10 is sufficient for direct activation of the effector caspases required for activating the apoptotic cascade. In type-II cells, the induction of apoptosis upon TRAIL-R triggering requires the amplification of the initial signal via engagement of the mitochondrial/intrinsic apoptosis pathway. In these cells, activated caspase-8 leads to Bax/Bak-mediated mitochondrial outer membrane permeabilization (MOMP) via truncated Bid^9^. Upon MOMP pro-apoptotic factors, most importantly cytochrome c, are released to the cytosol, the prerequisite for the formation of the Apoptosome. Within the Apoptosome caspase-9 is activated, which in turn is able to fully activate caspase-3 to trigger apoptosis in type-II cells. Importantly, PDAC cells have been shown to employ a type-II apoptotic signaling pathway upon death receptor stimulation^10^.

Intriguingly, TRAIL was found to be able to induce apoptosis in cancer cell lines in vitro and in vivo while sparing normal, healthy tissues^11,12^. Consequently, exploiting TRAIL for anticancer therapy was thought to represent a promising therapeutic strategy^11^. Within the following years, multiple TRAIL-receptor agonists (TRAs) were developed for clinical application. Recombinant TRAIL (Dulanermin) and several agonistic TRAIL-receptor-specific antibodies (e.g., Mapatumumab and Conatumumab) entered clinical trials^13^. These trials confirmed broad tolerability and safety of these agents in patients^14^. However, despite promising preclinical results, also in PDAC, none of the TRAs achieved a therapeutic effect in randomized-controlled clinical trials^15,16^. Of note, recent studies have demonstrated that TRAIL-receptor triggering may even enhance the invasive, proliferative and metastatic potential in cancer cells^17–19^. Consequently, in scenarios, in which TRAIL-R triggering is not capable of sufficiently activating the apoptotic cascade, the application of TRAs may promote cancer progression. Two major facts are currently thought to account for the fact that exploring TRAIL for anticancer therapy could so far not live up with the high expectations that arose from preclinical studies. First, it has become evident that in many cancer cells TRAs need to be combined with sensitizing agents to break resistance of cancer cells. Second, TRAs with superior agonistic activity need to be developed, since so far, TRAs with comparable low agonistic activity have entered clinical trials.

Recently, a novel TRA was designed to imitate the TRAIL-R1/TRAIL-R2 interaction sides of TRAIL. This agonistic fusion protein, named APG350, comprises two single-chain TRAIL-receptor binding domains covalently linked to the Fc-portion of a human IgG1 molecule. APG350 forms dimers in which each dimer binds to three TRAIL-receptors^20^. The resulting hexavalent binding mode enables clustering of six TRAIL-receptors and revealed superior apoptosis-inducing activity in a variety of human tumor cell lines and primary tumor cells, for example, colon, liver, breast and lung cancer compared with recombinant, untagged TRAIL^20^. Moreover, APG350 treatment reduced the size of subcutaneously implanted human colon carcinoma cell line Colo205 in mice. Considering these promising results and the urgent need for novel therapeutic options for PDAC, we set out to evaluate the effects of APG350 on PDAC cells both in vitro and in vivo, employing a recently developed clinically adapted orthotopic mouse xenotransplantation model of PDAC with and without primary tumor resection.

## Results

### Characterization of APG350-induced apoptotic and non-apoptotic signaling in PDAC cells

**Fig. 1:**
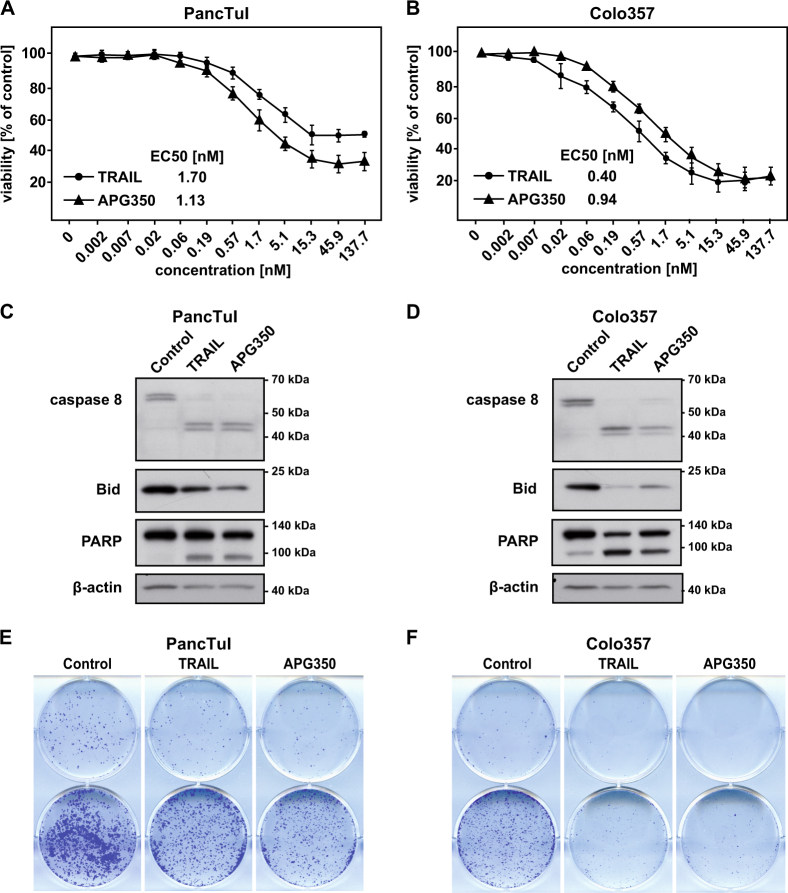
APG350 induces cell death in PDAC cells. PDAC cells were treated for 24 h with indicated concentrations of soluble TRAIL or APG350. Viability of PancTuI **a** or Colo357 **b** was determined using crystal violet staining. Values are means ± SD (*n* = 6). PancTuI **c** and Colo357 **d** were stimulated with TRAIL or APG350 (both in conc. 1.7 nM) for 24 h and the expression and cleavage of caspase-8, Bid and PARP were analyzed in whole-cell lysates by western blot. β-Actin was used as a gel-loading control. PancTuI **e** and Colo357 **f** cells were seeded into six-well plates with a density of 500 (upper part) or 5000 (lower part) cells/well, treated with 1.7 nM TRAIL or APG350 for 24 h and clonogenic survival was determined by crystal violet staining. Shown are representative results out of three independent experiments performed

APG350 has been shown to harbor superior antitumor activity over trimeric, untagged TRAIL on colon carcinoma cells^20^. To prove whether it holds true also for PDAC cells, we first compared side-by-side the effects of APG350 and recombinant TRAIL on PDAC cells in vitro. In agreement with our previous data, TRAIL treatment reduced viability of cells in a dose-dependent manner^21^. Treatment with APG350 also induced a potent cytotoxic effect in these cells (Figs. 1a, b and Suppl. Figure 1A). To directly compare the apoptotic activity of APG350 and TRAIL, we determined the EC50 based on the molar concentration. In PancTuI cells, APG350 exerted a superior cytotoxic activity compared with soluble TRAIL, whereas in Colo357 and Panc89 cells TRAIL treatment was more efficient at the same concentration (Figs. 1a, b and Suppl. Figure 1A). Both, APG350 and TRAIL efficiently induced cleavage of caspase-8, its target Bid and the target of caspase-3 PARP (Figs. 1c, d and Suppl. Figure 1B). Importantly, treatment of cells with TRAIL or APG350 for 24 h potently reduced clonogenic survival (Figs. 1e, f and Suppl. Figure 1C).

**Fig. 2:**
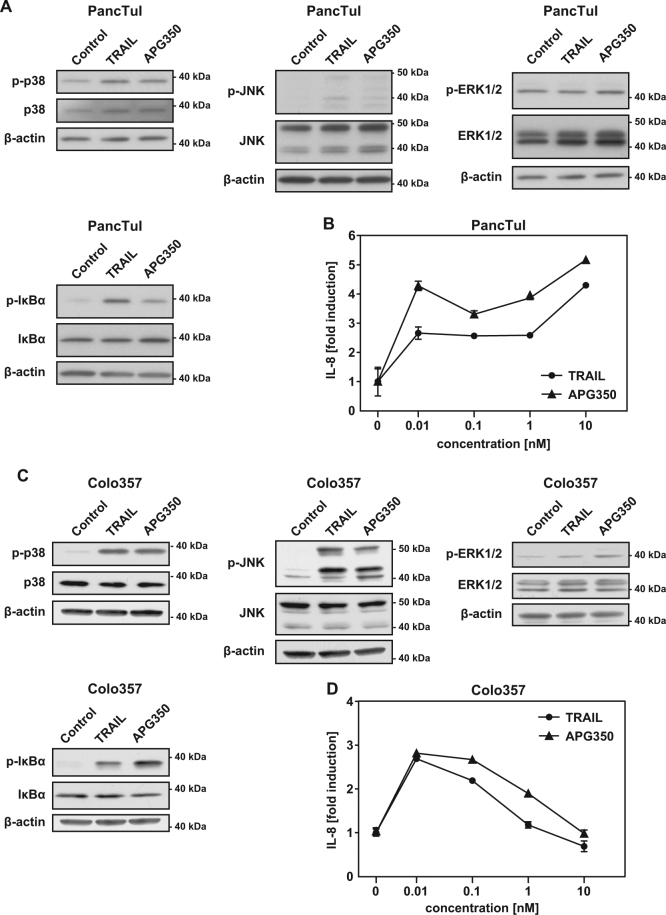
APG350 induces pro-inflammatory signaling in PDAC cells. PancTuI **a** and Colo357 **c** cells were treated with TRAIL or APG350 (both in conc. 1.7 nM) for 3 h. The phosphorylation of MAP kinases p38, p-JNK and ERK1/ERK2 as well as of IκBα was analyzed by western blotting in whole-cell lysates by usage of phospho-specific antibodies. As control, the total expression levels of corresponding proteins and the cellular levels of β-actin were determined in parallel. PancTuI **b** and Colo357 **d** cells were treated with indicated concentrations of TRAIL or APG350 for 24 h. IL-8 concentration in cell culture supernatants was determined by ELISA. Shown are means ± SD (*n* = 6)

Beside induction of apoptosis, triggering of TRAIL-Rs in PDAC cells may also engage pro-inflammatory signaling pathways leading to increased invasion and metastasis^19,22,23^. To study the pro-inflammatory potential of APG350, we analyzed the APG350- and TRAIL-induced phosphorylation/activation of mitogen-activated protein (MAP) kinases and of inhibitory κBα (IκBα) as a marker for nuclear factor-κB (NF-κB) activation. APG350 and TRAIL-induced phosphorylation of p38 and IκBα in both cell lines (Figs. 2a, c). In Colo357 cells, both agents also triggered activation of c-Jun N-terminal kinase (JNK) and extracellular-signal regulated kinase 1/2 (ERK1/ERK2). Moreover, APG350 and TRAIL induced secretion of the pro-inflammatory cytokine IL-8 (Figs. 2b, d). Similarly to our previous results obtained for recombinant, untagged TRAIL^24^, IL-8 induction was more pronounced in PancTuI than in Colo357 cells upon TRAIL and APG350 treatment (Figs. 2b, d).

### Effect of constitutively overexpressed Bcl-xL on APG350-induced apoptosis and pro-inflammatory response

**Fig. 3:**
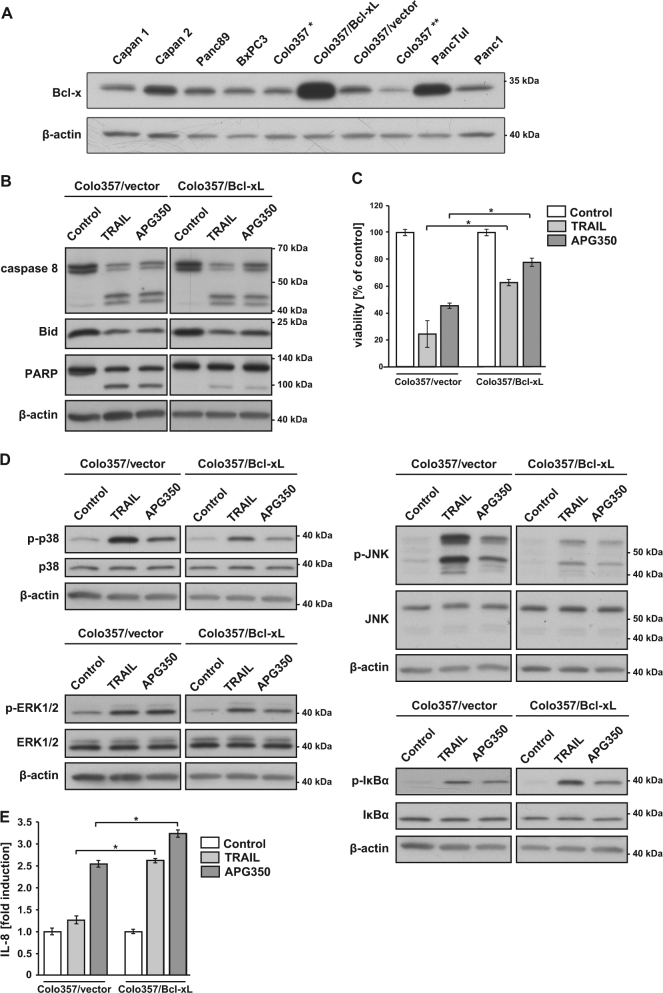
Overexpression of Bcl-xL influences apoptotic and pro-inflammatory APG350-induced signaling. **a** Whole-cell lysates of different PDAC cell lines were tested for the expression of Bcl-xL via western blot. *Colo357 wild type cells used for the generation of stable cell line-overexpressing Bcl-xL. **Another charge of Colo357 cells line. **b**–**d** Colo357 cells stably overexpressing Bcl-xL (Colo357/Bcl-xL) and the corresponding mock-transfected control cells (Colo357/vector) were treated with TRAIL (1.7 nM) or APG350 (1.7 nM) and the apoptotic and pro-inflammatory responses were analyzed. **b** Cells were treated for 24 h. Whole-cell lysates were analyzed for the expression and cleavage of caspase-8, Bid and PARP by western blot. The level of β-actin was determined in parallel and served as a gel-loading control. **c** Viability of the cells was determined by crystal violet staining. Values are means ± SD (*n* = 6). *p < 0,05. **d** Cells were treated for 3 h. Phosphorylation of MAP kinases and IκBα was analyzed by western blot using phosphorylation-status detecting antibodies. As an equal gel-loading control, the total levels of corresponding proteins as well as the level of β-actin were determined in parallel. **e** IL-8 concentration was determined by ELISA in cell culture supernatants of cells treated with TRAIL (1 nM) or APG350 (1 nM) for 24 h. Shown are means ± SD (*n* = 6)

PDAC cells constitutively express high levels of Bcl-xL, which in turn protects these cells from chemotherapy-induced apoptosis^25,26^. Moreover, elevated Bcl-xL expression mediated TRAIL resistance in these cells and enhanced non-apoptotic signaling at the same time^10,19^. To study whether increased Bcl-xL expression could also influence APG350-induced signaling, we stably transfected Colo357 cells with an expression vector encoding Bcl-xL. The expression level of Bcl-xL in different established PDAC cell lines and generated Colo357/Bcl-xL cells are shown in Fig. 3a. Next, we compared Colo357/Bcl-xL with the corresponding mock-transfected cells (Colo357/vector) in their response to APG350 treatment. Consistent with the known type-II mode of apoptosis induction in the parental Colo357 cells, overexpression of Bcl-xL had no significant influence on the APG350/TRAIL-induced cleavage of caspase-8 and Bid but diminished the cleavage of the downstream apoptotic target PARP (Fig. 3b). Intriguingly, Colo357/Bcl-xL cells were protected from both, TRAIL- and APG350-induced apoptosis (Fig. 3c). Analyses of the TRAIL-R-mediated activation of non-apoptotic signaling pathways revealed that Colo357/Bcl-xL compared with Colo357/vector responded to APG350/TRAIL with diminished activation of p38 and JNK, with comparable activation of ERK1/ERK2, and, in the case of TRAIL, with stronger activation of NF-κB (Fig. 3d). In both cell lines, TRAIL was a stronger activator of p38 and JNK than APG350. Importantly, TRAIL and APG350 stimulated the secretion of IL-8, the effect strongly enhanced by the overexpression of Bcl-xL (Fig. 3e). Of note, in both cell lines APG350 proved to be a stronger inducer of IL-8 than TRAIL.

### Effects of APG350 treatment on primary tumor growth (palliative treatment)

**Fig. 4:**
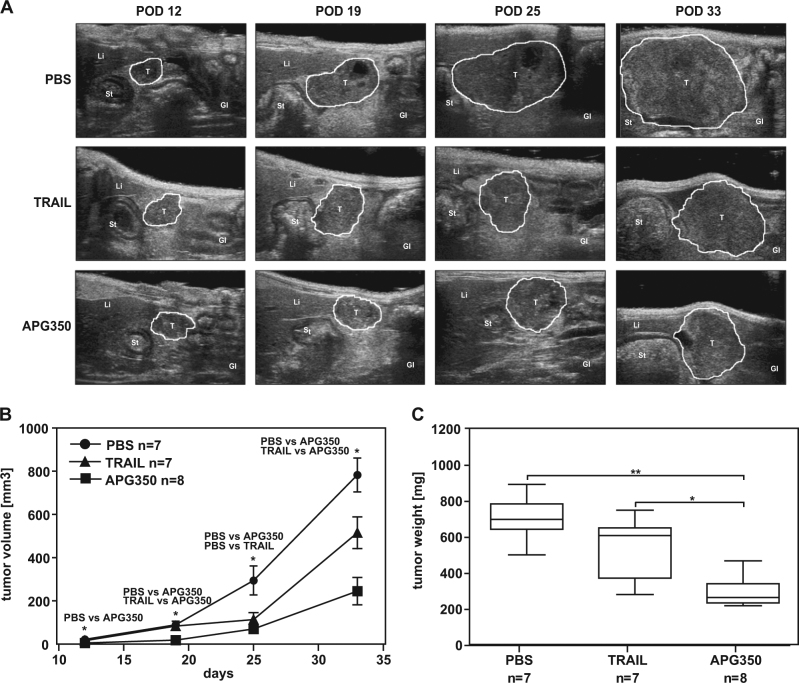
Effects of APG350 on primary tumor growth. PancTuI cells were orthotopically inoculated into SCID/beige mice. Four days later, mice were randomized and treated i.p. with TRAIL (3 mg/kg body weight), APG350 (3 mg/kg body weight) or PBS for 5 following days. The animals were sacrificed 33 days post op. **a** Tumor growth was monitored on day 12, 19, 25 and 33 post op by ultrasound imaging with mice in supine position and sagittal transducer orientation. Shown are representative time-course ultrasound images of one animal per group. **b** Tumor volume measured in 3D motion are shown as median with standard deviation. Significance was tested using the Mann–Whitney *U*-test, *p < 0.05. **c** Tumor weight is shown as box plots with median; top of each box, 75th percentile; bottom, 25th percentile. Significance was tested using the Mann–Whitney *U*-test, *p < 0.05, **p < 0.01

**Fig. 5:**
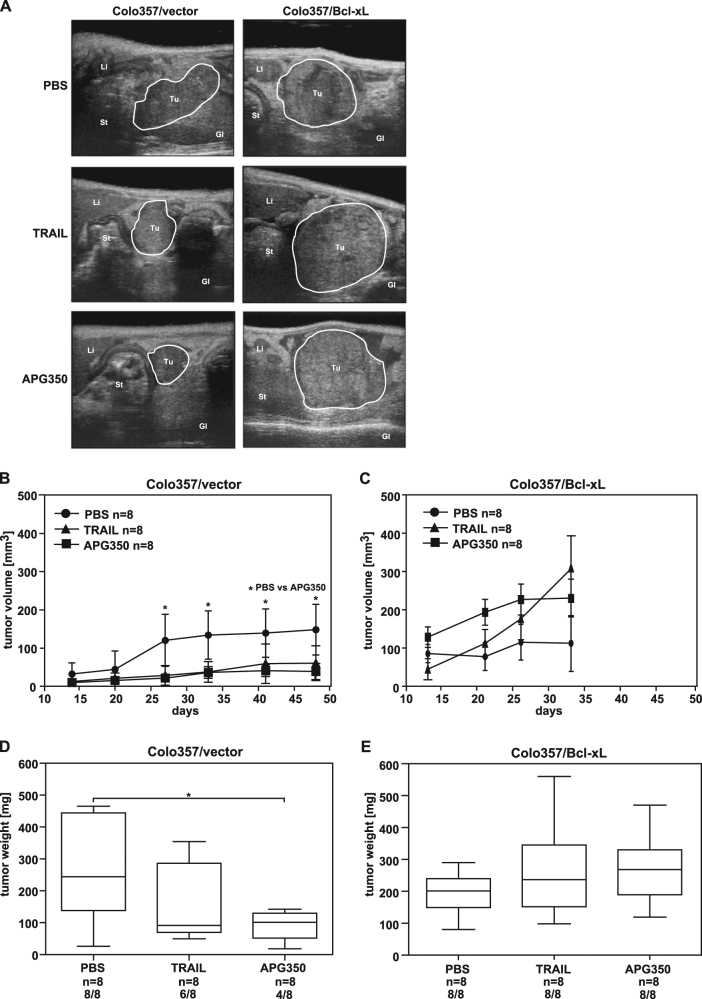
Effects of APG350 on primary tumor growth in Colo357 tumors with or without Bcl-xL overexpression. Colo357/vector or Colo357/Bcl-xL cells were orthotopically inoculated into SCID/beige mice. Four days later, mice were randomized and treated i.p. with TRAIL (3 mg/kg body weight), APG350 (3 mg/kg body weight) or with PBS for 5 following days. The animals were sacrificed 33 days post op (Colo357/Bcl-xL-bearing mice) or 50 days post op (Colo357/vector-bearing mice). **a** Representative images on day 33 after tumor cell inoculation are shown. **b**,** c** Tumor volumes measured in 3D motion are shown as median with standard deviation. Significance was tested using the Mann–Whitney* U*-test, *p < 0.05. **d**, **e** Tumor weight are shown as box plots with median; top of each box, 75th percentile; bottom, 25th percentile. Significance was tested using the Mann–Whitney *U*-test, *p < 0.05

Prompted by the in vitro results showing strong apoptosis-inducing capacity of APG350 on PDAC cells, we sought to determine whether it could also exert therapeutic capabilities in vivo. For this purpose, we employed an orthotopic PDAC xenotransplantation model^27^. In the first set-up, resembling a palliative treatment regime, mice with established orthotopically implanted tumors were treated with APG350 or TRAIL and the effects of each agent on primary tumor growth were analyzed. Treatment started at day 4 after tumor cell inoculation and mice were treated either with vehicle (phosphate-buffered saline, PBS), with APG350 or with TRAIL once per day i.p. on 5 consecutive days. As revealed by ultrasound and high-resolution three-dimensional (3D) volumetry, APG350 inhibited the growth of PancTuI-derived tumors leading to drastic and significant reduction of the tumor volume (Figs. 4a, b). Intriguingly, therapeutic efficiency of APG350 treatment was significantly superior to TRAIL treatment. Accordingly, tumor weight in mice treated with APG350 was strongly and significantly reduced compared with the PBS-treated control mice and to TRAIL-treated mice (Fig. 4c). Next, we set out to investigate the effect of Bcl-xL overexpression in this model. In line with the results obtained in vitro, tumors derived from Colo357/vector cells responded to APG350 and TRAIL treatment (Figs. 5a, b, d). Tumor volume (Fig. 5b), as well as tumor weight (Fig. 5d) were significantly and strongly decreased in APG350-treated mice. Importantly, in contrast, to the in vitro data (Fig. 3c), APG350 treatment was more effective than TRAIL treatment at the same administered dosage. Overexpression of Bcl-xL, however, conferred resistance to both, APG350 and TRAIL and treatment failed to reduce tumor burden (Figs. 5a, c, e). Interestingly though, despite lacking statistical significance, there was even a trend toward increased tumor burden upon treatment in these cells. Staining of tumor tissues with antibodies against the proliferation marker Ki67 revealed, however, no significant effect of APG350 or TRAIL on tumor cell proliferation (Suppl. Figure 2).

### Effects of APG350 treatment on tumor recurrence and metastasis upon tumor resection (adjuvant treatment)

**Fig. 6:**
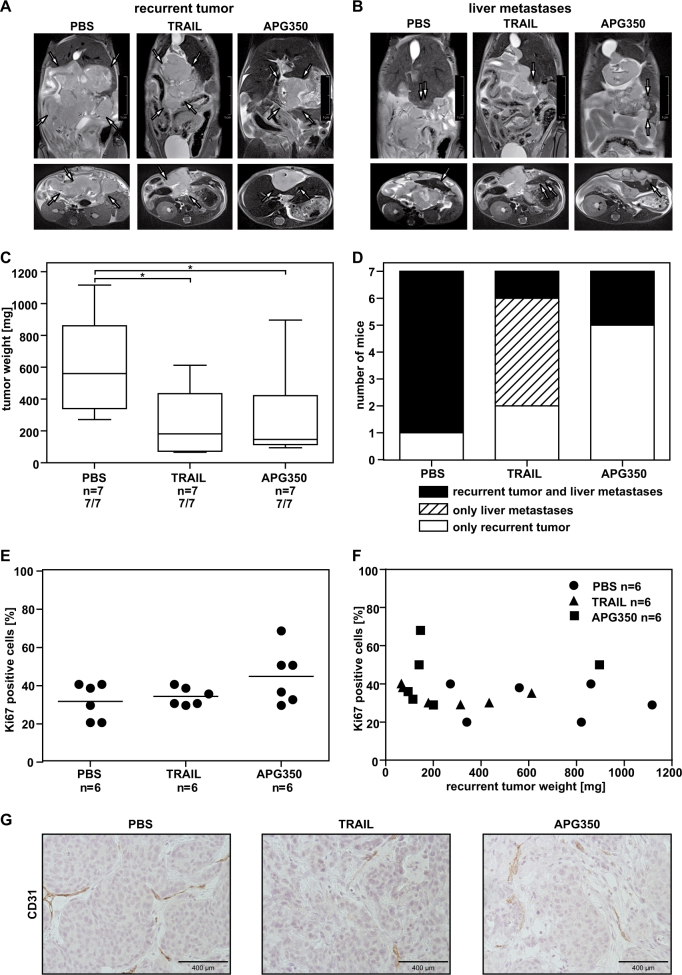
Effects of APG350 treatment on recurrent tumor growth and metastasis. PancTuI cells were injected orthotopically into pancreas of SCID bg mice. Fourteen days later primary tumor resection was performed via subtotal pancreatectomy. Four days later mice were randomized and treated i.p. with TRAIL (3 mg/kg body weight), APG350 (3 mg/kg body weight) or PBS for 5 consecutive days. On day of sacrifice (day 36), tumors were removed and weighed. Metastases were macroscopically identified and counted. Representative MRI scan pictures showing recurrent tumor growth **a** and liver metastases **b** in PBS-, TRAIL- or APG350-treated animals on day 22 after primary tumor resection. **c** Effect of TRAIL and APG350 on PancTuI-derived tumors. Recurrent tumor weight is shown as box plots with median; top of each box, 75th percentile; bottom of box, 25th percentile. Significance was tested by Mann–Whitney* U-*test, *p < 0.05. **d** Number of mice that developed recurrent tumor only or recurrent tumor and liver metastases. **e** Paraffin-embedded tumors were sectioned and stained with antibodies against Ki67. Each tumor was scanned and Ki67 positive cells were evaluated per 100 tumor cells. **f** Correlation between recurrent tumor weight and degree of proliferation as detected by counting Ki67-positive cells. **g** Images showed CD31 stained tumor cells in TRAIL, APG350- or PBS-treated tissue

Encouraged by the results obtained in the palliative setting, we set out to investigate whether APG350 could inhibit the recurrent tumor growth and/or metastasis. To address this question we inoculated PancTuI cells into murine pancreas, resected primary tumors by subtotal pancreatectomy and treated mice with vehicle, APG350 or TRAIL administrated intraperitoneally on 5 consecutive days. We monitored the recurrent tumor growth via ultrasound imaging and the onset of liver metastases via magnetic resonance imaging (MRI; Figs. 6a, b). Recurrent tumor weight and the number of macroscopic liver metastases were determined post mortem. Irrespective of the treatment, all mice developed local recurrent tumor. Importantly, however, APG350 and TRAIL treatment significantly and to a similar extend reduced the recurrent tumor burden (Fig. 6c). In addition, six of seven PBS-treated mice (86%) developed liver metastases (Fig. 6d). TRAIL treatment only marginally prevented metastatic spread, because liver metastases were detected in five of seven mice (71%). In contrast, only two of seven mice (29%) treated with APG350 presented with liver metastases. Immunohistological analyses of tumor tissues with antibodies against the proliferation and blood vessel markers Ki67 and CD31, respectively, revealed no statistically significant effect of APG350 or TRAIL on tumor cell proliferation or neo-angiogenesis (Figs. 6e–g).

### Effects of combination of BH3-mimetics and APG350 on viability of PDAC cells

**Fig. 7:**
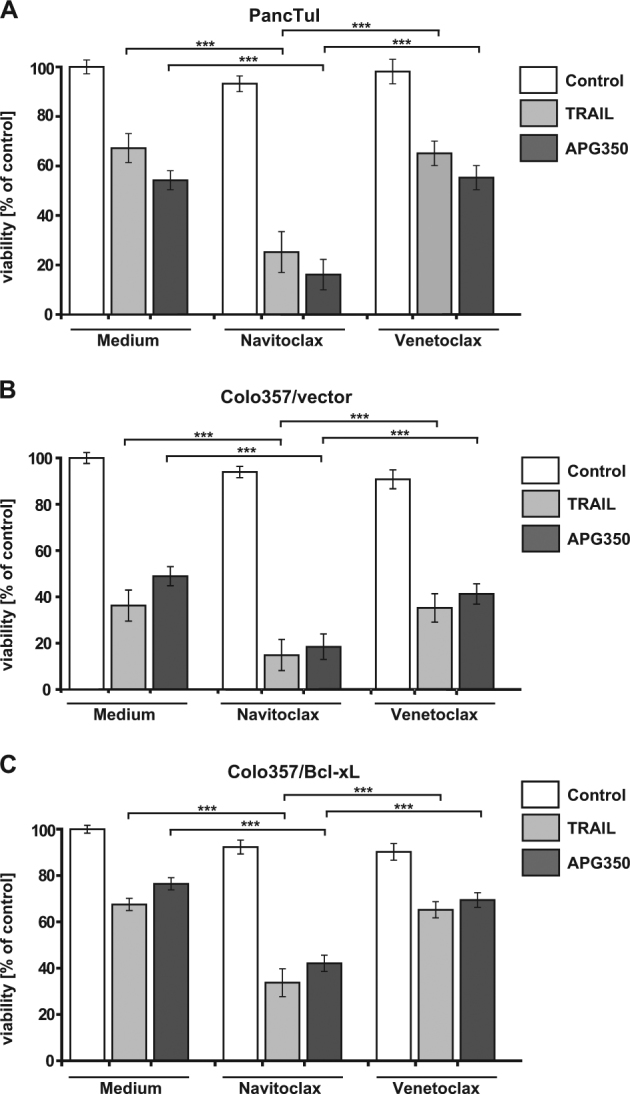
Effects of a combination of BH3-mimetics with TRAIL or APG350 on PDAC cell viability. PancTuI **a**, Colo357/vector **b** and Colo357/Bcl-xL **c** were pretreated with Navitoclax or Venetoclax (both in conc. 5 µM) for 2 h and stimulated for additional 24 h with TRAIL or APG350 (both in conc. 1.7 nM). Cell viability was determined using crystal violet staining. Values are means ± SD of four independent experiments *n* = 6. ***p < 0.001

Our results revealed that APG350 exerts broad apoptotic activity in PDAC. However, block of the mitochondrial apoptotic pathway by overexpression of Bcl-xL prevented APG350′s therapeutic efficacy in a palliative setting (Fig. 5). Recently, so called BH3-mimetics have been developed, which bind to and neutralize the activity of anti-apoptotic members of the Bcl-2-family. Among several generated and pre-clinically evaluated BH3-mimetics, Navitoclax (ABT-263) and Venetoclax (ABT-199) have successfully entered clinical testing^28^. Navitoclax potently antagonizes Bcl-2 and Bcl-xL, whereas Venetoclax selectively inhibits Bcl-2. We set out to investigate, whether BH3-mimetics may harbor the potential to reverse the counteracting effect of Bcl-xL overexpression on the APG350′s activity in PDAC cells. In PancTuI, Colo357/vector and Panc89 cells, Navitoclax potently sensitized to TRAIL- and APG350-induced apoptosis (Figs. 7a, b and Suppl. Figure 3). In contrast, Venetoclax only marginally, if at all, enhanced TRAIL/APG350-induced apoptosis, highlighting the pivotal role of Bcl-xL in mediating apoptosis resistance in PDAC cells. Even more importantly, however, Navitoclax, was capable to restore apoptosis induction by APG350 and TRAIL in Colo357/Bcl-xL cells (Fig. 7c).

## Discussion

Complete surgical resection provides the only chance for cure in PDAC. However, this option can be pursued in only 10–15% of all patients because PDAC is mostly diagnosed at a locally advanced or metastatic tumor stage^29^. Moreover, even when complete resection of the primary tumor is achieved, the majority of patients develop local recurrent disease and/or metachronous metastases and die shortly after^30,31^. These alarming facts suggest the existence of local tumor cell dissemination and distant (micro)metastases already at the time of initial diagnosis. To prolong progression-free survival, adjuvant chemotherapy is recommended for PDAC patients after tumor resection. Currently, gemcitabine or 5-fluorouracil (5-FU)-based therapies are mostly applied^32,33^, but their efficacies in improving overall and disease-free survival are limited^34,35^. In patients with primarily non-resectable PDAC, distant metastases or tumor recurrence, chemotherapeutic agents are employed within a palliative treatment approach to prolong survival and alleviate tumor-associated morbidity. Although with Folfirinox, an improvement of palliative treatment has recently been achieved, only selected patients may benefit from this treatment, and in addition, the treatment is associated with severe side effects^36^. Conventional chemotherapeutic agents induce DNA damages, which activate the intrinsic apoptotic pathway via the tumor-suppressor p53 and as recently shown by p63 and p72^37,38^. However, p53 coding gene is commonly mutated in PDAC leading to loss of expression or functional inactivation conferring PDAC cells widely resistant to these therapies^39^. These facts emphasize the urgent need for the development of novel treatment modalities to effectively and selectively kill PDAC cells by circumventing chemoresistance of these cells.

The identification of the death ligand TRAIL, has initially raised great expectations, because TRAIL was shown to induce apoptosis selectively in cancer cells without causing systemic or hepatic toxicity in particular^11^. Moreover, TRAIL induces apoptosis independently of p53^40^ and may therefore also be effective in p53-mutated, chemoresistant cancers such as PDAC. Giving the promising preclinical results TRAs, comprising soluble, untagged TRAIL and agonistic TRAIL-receptor-specific antibodies were developed for clinical application. However, the results of clinical trials have so far been disappointing. Recent studies have identified several pitfalls of current TRAIL-based treatment, among them the resistance of many primary tumor cells against TRAIL-mediated apoptosis^13,41,42^. An obvious strategy to overcome this resistance is based on combination of TRAs with target-specific sensitizing drugs. Thus, several, promising sensitizing strategies have recently be evolved^43^.

Another explanation for the failure of TRA in clinical trials might relate to the fact that currently employed TRAs may insufficiently induce apoptosis because of insufficient clustering of the targeted receptors^44^. In line with this consideration, recent studies have demonstrated that agonistic antibodies targeting TRAIL-Rs require further cross-linking to exhibit apoptotic activity^45^. Moreover, the combination of recombinant TRAIL with the agonistic anti-TRAIL-R2 antibody increased receptor clustering leading thereby to efficient apoptosis induction^46,47^. To increase the apoptosis-inducing potency, several recombinant TRAIL agonists were developed by linking TRAIL with different tags such as polyhistidine (His), leucine Zipper (LZ), isoleucine Zipper (IZ) and Flag^48,49^. However, due to hepatotoxicity induced by higher aggregated and crosslinked TRAIL formulation, soluble TRAIL and agonistic antibodies were selected for clinical development at this time^48,49^. A more sophisticated approach for enhancing the ligand activity may be achieved by construction of stable higher-order complexes of TRAIL by fusing it with a bridging molecule. APG350 represent a novel example of such multimerized TRAIL molecule. APG350 is a fully human fusion protein in which two trimers of the extracellular domain of TRAIL are linked to Fc-part of a human IgG1 molecule, the modification leading to an enhanced receptor clustering. This hexavalent TRA potently induced apoptosis in cancer cell lines and primary cancer cells in vitro as well as in vivo in a subcutaneous Colo205 xenograft model^20^. APG350 showed a broad tolerability without dose-limiting toxicity when systemically used in mice or monkeys^20^. Importantly, no liver toxicity was observed.

Giving these encouraging results, we set out to evaluate APG350′s therapeutic capacity for PDAC. Since recent studies, including our own, have revealed ambivalent activities of TRAs in PDAC cells, we decided to explore the potential of APG350 in activating canonical and non-canonical TRAIL-receptor signaling pathways in PDAC cells side-by-side. APG350 induced apoptosis in all established PDAC cell lines tested. Gieffers et al. showed that APG350′s apoptotic activity was superior to soluble TRAIL when treating cancer cells lines of different origin^20^. We found that in vitro some PDAC cell lines (Colo357, Panc89) are more susceptible to TRAIL- than to APG350-induced cell death, whereas others (PancTuI) respond other way round. With respect to these results, it has been shown previously, that, unlike most other tumor cells, PDAC cells preferentially induce apoptosis via TRAIL-R1^22^. Thus, the superior activity of TRAIL in Colo357 and Panc89 in vitro might relate to this finding. Recently, APG350 was successfully tested in a subcutaneous mouse model using colon carcinoma cell line^20^. However, it is widely accepted that tumor microenvironment has an influence on the growth of tumor cells, as well as on their sensitivity to therapeutic drugs^50^. This particularly applies for stroma-rich tumors like PDAC. Consequently, to study the therapeutic potential of APG350 for PDAC, we applied an orthotopic mouse xenotransplantation model. We evaluated the effects of APG350 treatment on primary tumor growth and, in addition, on recurrent tumor growth and metastasis following primary tumor resection. Clinical characteristics such as primary infiltrating tumor growth, local recurrence and distant metastases upon tumor resection mimic the clinical setting in these models^51^. Importantly, we observed a significant reduction of primary tumor size by APG350 treatment. Moreover, APG350 treatment potently suppressed local recurrent tumor growth and metastasis upon surgical resection. Of note, in both in vivo models APG350′s therapeutic activity was more effective than treatment with soluble TRAIL at the same dosage. As mentioned above, our in vitro studies revealed superior activity of APG350 in PancTuI but not in Colo357 and Panc89 cells. However, intriguingly, APG350 activity was superior to TRAIL in all in vivo models tested, including orthopically implanted Colo357 cells. APG350 represent a novel fusion protein highly diverse to soluble TRAIL with respect to its molecular structure. These differences determine differences in pharmacokinetic and pharmacodynamic characteristics that certainly emerge in in vivo scenarios. Based on the discrepancy of APG350′s activity in vitro vs. in vivo, we emphasize that these revised pharmacokinetic properties play a pivotal role for APG350 therapeutic supremacy. In summary, our findings demonstrate therapeutic potential of APG350 within palliative and adjuvant therapeutic strategies in PDAC. Importantly, APG350 treatment did not influence proliferation and/or angiogenesis in vivo, highlighting that its therapeutic activity is based on apoptosis-inducing capacity.

Our previous studies have highlighted the potential risk of TRAIL-comprising therapies in TRAIL-resistant PDAC cells due to the activation of non-canonical TRAIL signaling, promoting invasion and metastases^19,21,22^. Similar results have been demonstrated for other tumor entities^18,52^. In this respect, our current study show that also APG350 can activate MAPKs and NF-κB in PDAC cells and induces the secretion of pro-inflammatory cytokine IL-8. These effects were amplified in cells harboring resistance due to the overexpression of Bcl-xL. These results suggest that pro-inflammatory activities and potential adverse, tumor-promoting effects should be taken into account when applying APG350.

Cancer cells are often intrinsically resistant to conventional chemotherapeutic agents and also to death ligands including TRAIL. One major player in this context is Bcl-xL, in particular regarding PDAC cells, in which Bcl-xL overexpression is frequently detected^26^. Stable overexpression of Bcl-xL in apoptosis sensitive PDAC cell line Colo357 represents an experimental model to elucidate the impact of high and low Bcl-xL-levels on the response of cells to therapeutic agents. Intriguingly, we found that apoptosis was significantly diminished in Colo357/Bcl-xL cells compared with control cells revealing that enforced clustering of TRAIL-receptors by hexavalent ligands alone does not override mitochondrial blockade of apoptotic signaling. Nevertheless, apoptosis induction by APG350 was restored when BH3-mimetic Navitoclax was employed in combination. These results are in agreement with recently demonstrated potential of Navitoclax to sensitize different PDAC cell lines to soluble TRAIL^53^. Our present data suggest that APG350-comprising therapies can be effectively applied also in patients that acquired mitochondrial apoptosis resistance. However, it needs to be considered that besides overexpressing Bcl-2 proteins, the overexpression of additional anti-apoptotic (or loss of pro-apoptotic) factors can inhibit apoptosis in all steps of the apoptotic cascade. Therefore, it will be fundamental to evaluate APG350 in combination with additional agents, which modulate apoptotic sensitivity in a different mode of action.

In summary, our study provides a comprehensive analysis of the biological activity of APG350 in preclinical models for PDAC. We could clearly demonstrate the therapeutic potential of this compound administered in a palliative and in an adjuvant setting in a clinically adapted orthotopic mouse xenotransplantation model of PDAC. These encouraging results suggest that TRAIL-R agonists based on the hexavalent structural concept represent a very promising novel, next-generation TRA for the treatment of PDAC. However, their potential to activate non-apoptotic signaling and apoptosis resistance mechanisms operating in PDAC cells demand additional preclinical and clinical studies to further elucidate their therapeutic efficiency for this tumor entity.

## Materials and methods

### Cell lines and culture conditions

Human pancreatic adenocarcinoma cell lines Colo357, PancTuI, Panc89 and their suppliers have been described previously^54^. Bcl-xL-overexpressing Colo357 and control cells were established by transduction of Colo357 cells with a retroviral Bcl-xL-encoding expression vector pBabe-puro or a control vector, respectively, according to the protocol provided in Hinz et al.^10^. All cells were grown in RPMI-1640 supplemented with 10% fetal calf serum (PAN-Biotech, Aidenbach, Germany), 2 mM glutaMAX and 1 mM sodium pyruvate (both from Life Technologies, Eggenstein, Germany) under standardized condition (5% CO_2;_ 37 °C). Recombinant cells were passaged in medium containing 2 µg/mL puromycin (Sigma-Aldrich, Darmstadt, Germany). APG350 was kindly provided by Apogenix AG (Heidelberg, Germany) and generated as described previously^20^. TRAIL was purchased from Peprotech, Hamburg, Germany. Navitoclax (ABT-263) and Venetoclax (ABT-199), both from Selleckchem, Biozol, Eching, Germany, were used in the concentration of 5 µM.

### Long-term survival

Long-term survival was analyzed as described previously^55^. Briefly, 500 or 5000 cells were seeded in six-well plates and 48 h later treated with TRAIL (1.7 nM) or APG350 (1.7 nM) for 24 h. Then cells were washed and cultured with complete medium for additional 5 days. Viable cell were visualized by staining with crystal violet (Sigma-Aldrich®; 1% crystal violet in 50% ethanol).

### Cell viability assays

Cells were seeded (1 × 10^6^/well) in 96-well plates, grown for 24 h and stimulated with different concentrations of TRAIL or APG350 for additional 24 h. Cell viability was determined by crystal violet staining as described previously^22^.

### IL-8 ELISA

For determination of IL-8 concentration in cell culture supernatants, an IL-8-Immunoassay (R&D Systems, Minneapolis, USA) was used according to the manufacturer’s protocol.

### Western blot analysis

Cells were seeded in six-well plates (2.5 × 10^5^/well for 3-h treatment; 2 × 10^5^/well for 24-h treatment), allowed to adhere for 24 h and treated with TRAIL or APG350 for either 3 or 24 h. Whole-cell lysates were prepared using RIPA buffer and analyzed by western blot as described^23^. Primary and secondary antibodies used were purchased from: Cell Signaling Technology, Frankfurt/ Main, Germany (anti-caspase-8, anti-PARP, anti-phospho-p38, anti-phospho-IκBα, anti-phospho-p42/44, anti-phospho-JNK, anti-p38, anti-p42/44, anti-JNK, anti-mouse-IgG-HRP, anti-rabbit-IgG-HRP), BD Bioscience, Heidelberg, Germany (anti-Bcl-xL), R&D Systems, Minneapolis, Canada (anti-Bid), Santa Cruz, Heidelberg, Germany (anti-IκBa, anti-goat-IgG-HRP) and Sigma-Aldrich (anti-β-actin).

### Laboratory animals

Female 6-week-old SCID bg mice (CB17.Cg-Prkdc^scid^Lyst^bg-J^/Crl) were purchased from Charles River (Sulzfeld, Germany). All animals housed in a sterile environment in individually ventilated cages (IVC) with access to water and food ad libitum and were allowed to acclimatize for 10 days before experiments started. The experiments were carried out in accordance with animal welfare and the local authorities (V 311-72241.121-7 (52-4/12)).

### Orthotopic xenotransplantation of human PDAC cells and palliative treatment

Orthotopic inoculation of tumor cells was performed as described previously^27^. Animals were randomized and palliative treatment started 4 days after tumor cell injection. TRAIL (3 mg/kg body weight), APG350 (3 mg/kg body weight) or PBS was injected i.p. on 5 consecutive days once per day. At the end of an experiment, mice were sacrificed, size and weight of each tumor was measured and metastases were documented.

### Relaparotomy, tumor resection and adjuvant treatment

The experimental set-up started as described above. Fourteen days after tumor cell inoculation, the tumors bearing pancreata were removed by a pancreatic-tail-resection as described previously^27,33^. Thereafter, starting on day 4 after primary tumor resection, mice were treated with TRAIL or APG350 (each 3 mg/kg body weight; i.p. once per day) or PBS on 5 consecutive days. Animals were sacrificed and examined for macroscopic metastases in liver and spleens and size and weight of recurrent tumors were documented.

### Ultrasound monitoring

Ultrasound imaging was performed with a Vevo2100 device (VisualSonics Inc., Toronto, Canada) equipped with a MS-550S transducer with a center frequency of 40 MHz. Automated 3D stacks of the tumors were obtained weekly after surgery in sagittal orientation with a slice interval of 100 µm. Tumors were identified on ultrasound images as hypoechogenic, irregularly shaped structures caudal to the liver, adjacent to the stomach and the duodenum. Blinded analysis was performed by semi-automated volumetry. For this purpose, the tumors were manually outlined on every fifth slice, and the interval of 500 µm was interpolated.

### Magnetic resonance imaging

MRI was performed at a magnetic field strength of 7 Tesla (ClinScan, Bruker Biospin, Ettlingen, Germany) using a four-channel phased-array coil for signal reception and a birdcage resonator (inner diameter 70 mm) for excitation (Bruker Biospin). Measurements comprised T2-weighted images of the abdomen (2D BLADE, TE/TR = 35/3310 ms, in-plane resolution 125 × 125 µm^2^, slice thickness 400 µm, 30 slices, matrix size 320 × 320) obtained in axial, coronal and sagittal orientation.

### Immunohistochemistry

Two µm sections of formalin-fixed and paraffin-embedded tissue samples were placed on slides and stained using antibodies directed against Ki67 (Dako, Glostrup, Denmark) or against CD31 (clone SZ31, Dianova, Hamburg, Germany), according to the protocol described in Goumas et al.^56^.

### Statistical analysis

Statistical analyses of the in vitro data were performed with GraphPad Prism Software 4.0 (La Jolla, CA) and comparison among groups was made by unpaired *t*-test. *p*-Values < 0.05 were considered as statistically significant. In vivo data were analyzed using SPSS 18.0 (SPSS Inc., Chicago, IL, USA). Owing to skewed data distribution (tested by Shapiro–Wilk test) different groups were analyzed non-parametrically by Mann–Whitney *U*-test. Differences were considered statistically significant at a level of *p* < 0.05.

## Electronic supplementary material

Supplementary Fig. 1

Supplementary Fig. 2

Supplementary Fig. 3

Supplementary Figure legends
