# Supplementary figures and images for: The novel TRAIL-receptor agonist APG350 exerts superior therapeutic activity in pancreatic cancer cells
Source: Cell Death Dis. 2018 Apr 18;9(5):445. doi: 10.1038/s41419-018-0478-0 (PMC5906476; doi:10.1038/s41419-018-0478-0)

Supplementary Fig.1

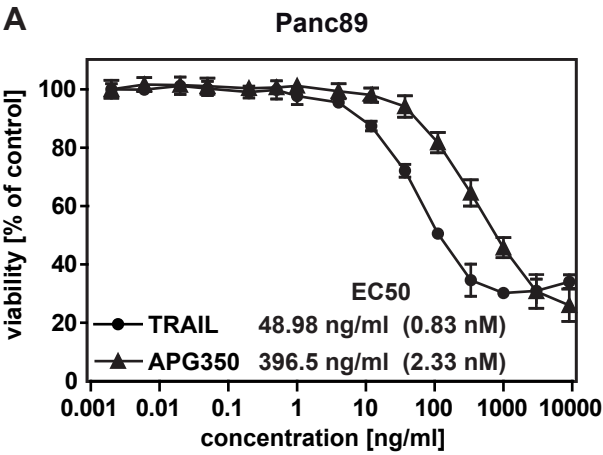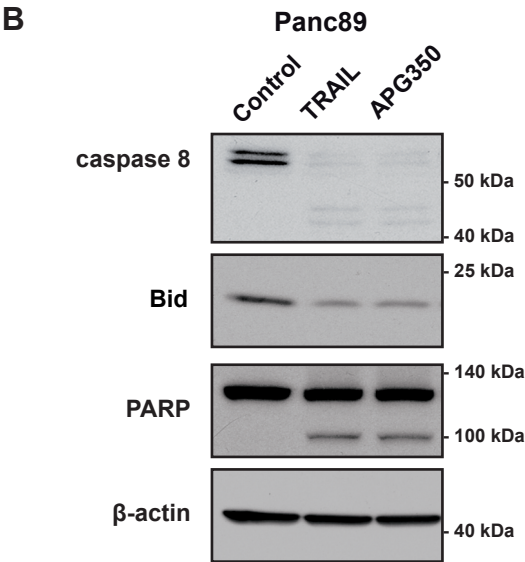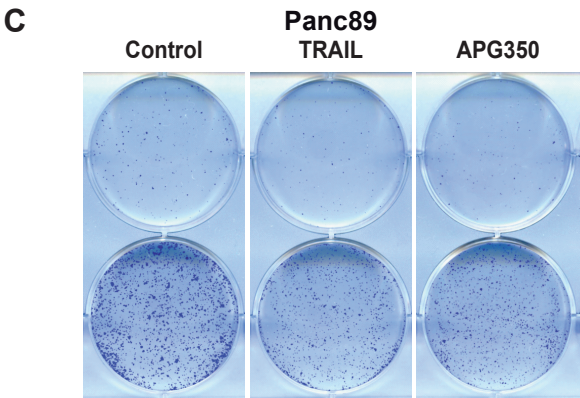

Supplement: Supplementary file 1 — Supplementary Fig. 1 [file 41419_2018_478_MOESM1_ESM.pdf]

Supplementary Fig. 2

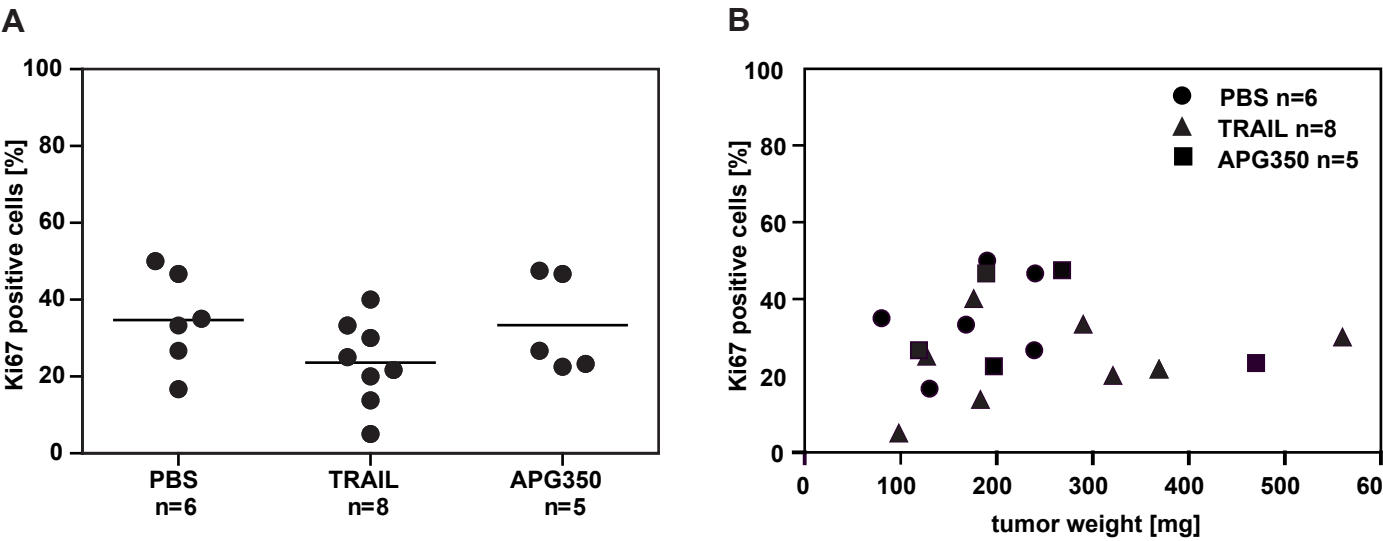

Supplement: Supplementary file 2 — Supplementary Fig. 2 [file 41419_2018_478_MOESM2_ESM.pdf]

Supplementary Fig. 3

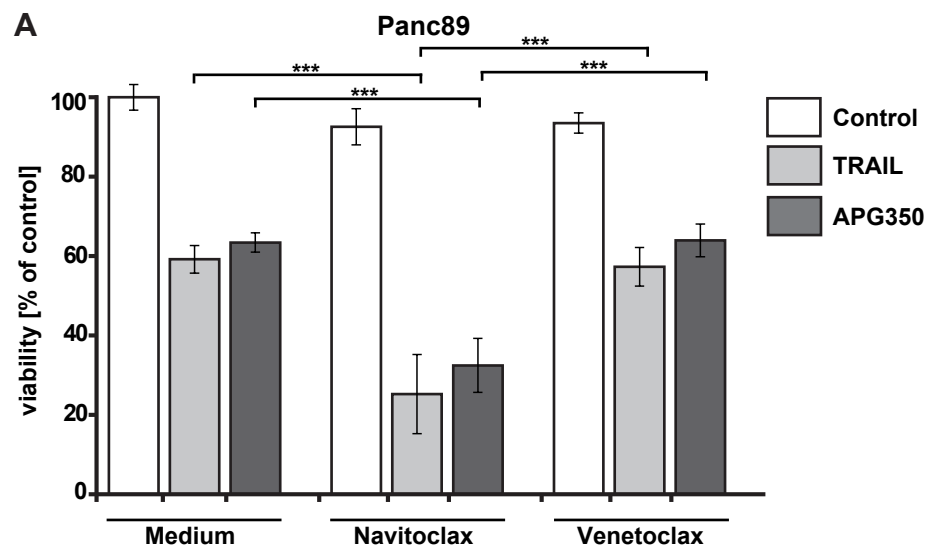

Supplement: Supplementary file 3 — Supplementary Fig. 3 [file 41419_2018_478_MOESM3_ESM.pdf]
