# Supplementary material for: The novel TRAIL-receptor agonist APG350 exerts superior therapeutic activity in pancreatic cancer cells
Source: Cell Death Dis. 2018 Apr 18;9(5):445. doi: 10.1038/s41419-018-0478-0 (PMC5906476; doi:10.1038/s41419-018-0478-0)
Supplement: Supplementary file 4 — Supplementary Figure legends [file 41419_2018_478_MOESM4_ESM.docx]

**Supplementary Figure Legends**

**Supplementary Figure 1: APG350 induces cell death in Panc89 cells.**

Panc89 cells were treated for 24 h with indicated concentrations of TRAIL or APG350. (A) Cell Viability was determined using crystal violet staining. (B) Cells were stimulated with TRAIL or APG350 (both in conc. 1.7 nM) for 24 h and the expression and cleavage of caspase 8, Bid and PARP were analyzed in whole cell lysates by Western blot. β-actin was used as a gel loading control. Values are means ± SD (n=6). (C) Cells were seeded into 6-well plates with a density of 500 (upper part) or 5000 (lower part) cells/well, treated with 1.7 nM TRAIL or APG350 for 24 h and clonogenic survival was determined by crystal violet staining. Shown are representative results out of four independent experiments performed.

**Supplementary Figure 2: Effects of APG350 on primary tumor growth in Colo357 tumors with or without Bcl-xL overexpression.**

Colo357/vector or Colo357/Bcl-xL cells were orthotopically inoculated into SCID/beige mice. Four days later mice were randomized and treated i.p. with TRAIL (3mg/kg body weight), APG350 (3mg/kg body weight) or with PBS for five following days. The animals were sacrificed 33 days post op (Colo357/Bcl-xL-bearing mice) or 50 days post op (Colo357/vector-bearing mice). Paraffin-embedded tumors were sectioned and stained with antibodies against Ki67. A) Each tumor was scanned and Ki67 positive cells were evaluated per 100 tumor cells. (B) Correlation between tumor weight and degree of proliferation as detected by counting the Ki67 positive cells.

**Supplementary Figure 3:** **Effects of a combination of BH3-mimetics with TRAIL or APG350 on the viability of Panc89 cells.**

Panc89 cells were pretreated for 2 h with Navitoclax (5 µM) or Venetoclax (5µM) and stimulated for additional 24 h with TRAIL (1.7 nM) or APG350 (1.7 nM). Cell viability was determined using crystal violet staining. Values are means ± SD of three independent experiments n=6. (***) p < 0.001.
